# Association between Psychological Factors and Condom Use with Regular and Nonregular Male Sexual Partners among Chinese MSM: A Quantitative Study Based on the Health Belief Model

**DOI:** 10.1155/2020/5807162

**Published:** 2020-09-28

**Authors:** Yuling Huang, Bin Yu, Peng Jia, Zixin Wang, Shifan Yang, Chunhua Tian, Wenhong Lai, Shujuan Yang

**Affiliations:** ^1^Center for AIDS/STD Control and Prevention, Sichuan Center for Disease Control and Prevention, Chengdu 610041, China; ^2^West China School of Public Health and West China Fourth Hospital, Sichuan University, Chengdu 610041, China; ^3^Department of Land Surveying and Geo-Informatics, The Hong Kong Polytechnic University, Hong Kong, China; ^4^International Institute of Spatial Lifecourse Epidemiology (ISLE), China; ^5^Centre for Health Behaviours Research, The Jockey Club School of Public Health and Primary Care, The Chinese University of Hong Kong, China

## Abstract

**Objective:**

The Chinese men who have sex with men (MSM) population is suffering from a high HIV infection rate owing to unprotected anal sex. The Health Belief Model (HBM) has been proven to be an effective frame associated with behavior maintenance. Based on HBM, we analyzed the beliefs associated with consistent condom use behavior with regular and nonregular partners among MSM to better provide targeted interventions and services.

**Methods:**

A study was conducted in Sichuan Province, China, from November 2018 to April 2019, and 801 eligible participants were recruited by snowball sampling. Sociodemographic characteristics, AIDS-related characteristics, sexual behaviors, condom use behavior, and dimensions of HBM were investigated. Univariate, single multivariate, and summary multivariate models were employed to analyze the factors associated with consistent condom use.

**Results:**

Of all participants, 39.1% and 53.6% had had anal sex with regular and nonregular partners in the last six months, respectively. Only 56.5% of them had used condoms consistently with regular partners, and only 60% of them had used condoms consistently with nonregular partners. When taking consistent condoms use with regular partners as the dependent variable, the dimensions of perceived threats (ORM = 1.28, 95% CI: 1.10, 1.49), perceived barriers (ORM = 0.70, 95% CI: 0.60, 0.82), self-efficacy (ORM = 1.23, 95% CI: 1.14, 1.32), and cues to action (ORM = 1.21, 95% CI: 1.02, 1.43) showed significant associations with the dependent variable. When taking consistent condoms use with nonregular partners as the dependent variable, the dimensions of perceived barriers (ORM = 0.77, 95% CI: 0.67, 0.89), self-efficacy (ORM = 1.22, 95% CI: 1.13, 1.32), and cues to action (ORM = 1.53, 95% CI: 1.30, 1.80) showed significant associations with the dependent variable.

**Conclusions:**

More attention should be focused on how to decrease the obstructive factors of condom use, how to improve the confidence of condom use, and how to layout more cues to action to promote consistent condom use behavior with regular and nonregular partners during anal sex among Chinese MSM.

## 1. Introduction

According to the annual report of HIV/AIDS cases in China, the composition ratio of sexual transmission owing to men who have sex with men (MSM) had increased from 3.4% (2007) to 28.2% (2015) and had stabilized at 28% during 2016 and 2017 [1]. The high HIV infection rate among MSM was ascribed to high-risk sexual behaviors, such as unprotected anal intercourse (UAI) and inconsistent condom use, through which HIV spread from HIV-positive MSM to general MSM [2, 3].

Although treatment as a means of prevention has been verified as efficacious [4], the unobserved HIV infections complicate the transmission network [5]. Those who have sex with partners of unknown HIV status might face the risk of infection. For the general MSM population, condom use still seems to be a crucial strategy for protection between partners. Having sex with regular male partners (e.g., couples with long-term intimate relationships) and nonregular male partners (e.g., partners for only one night) are two main types of sexual behavior among MSM, of which condom use is of concern [6]. However, according to a previous study, the prevalence of condom use with regular male partners and nonregular male sexual partners was only 55% and 66%, respectively, among Chinese MSM, resulting in the spread of HIV among this vulnerable population [7].

Recently, belief factors have been verified as the crucial psychological factors associated with condom use behavior among MSM [8, 9]. The psychological frame of the health belief model (HBM) has been proven associated with condom use behavior of anal sex among MSM in a previous qualitative study [10]. Specifically, HBM is a theoretical model, which was developed in the 1950s by social psychologists and was conceptualized by Rosenstock et al. in 1974 to explain the effect of the individual psychological course on the behavior change of maintenance [11]. Five constructs are comprised in the model: (1) perceived threats: the beliefs related to perceived susceptibility and perceived severity of poor health outcomes (e.g., disease). (2) Perceived benefits: the belief related to rewards and gain after adopting certain behavior. (3) Perceived barriers: the belief related to barriers and loss after adopting certain behavior. (4) Cues to action: external and internal cues that can trigger specific health-related behaviors. (5) Self-efficacy: confidence in certain behavior maintenance even in an adverse situation [11]. Previous studies have proven the effectiveness of HBM as a guidance framework among diverse health-related behaviors, such as mediation adherence [12], cancer screening [13], and condom use [14]. Based on the findings of which dimension of HBM is associated with health-related behavior change or maintenance, targeted interventions can be provided to achieve better health behaviors among vulnerable populations [6].

Currently, a limited number of studies related to HBM have been used to analyze the association between psychological factors and condom use behavior among MSM in China. A qualitative study carried out in China proved that the HBM could be applied to Chinese MSM and could provide information to help develop a population- and disease-specific HBM scale [10]. However, the qualitative evidence was insufficient to explain the specific effect of each dimension of HBM on condom use behavior. Furthermore, HBM-based behavioral interventions lack specific directionality. To further elaborate on the effect and directionality, an immediately followed study based on HBM was carried out in Chengdu, China [6]. The results showed that HBM-based interventions are warranted and should be designed with consideration of multidimensional factors and be partner type-specific [6]. However, it only focused on newly diagnosed HIV-positive MSM rather than the general MSM population. Moreover, since the general MSM population is vulnerable, condom use among which is also of great concern. Compared to HIV-positive MSM, general MSM might show different aspects of decision making regarding the use of condoms and responsibility for initiating safer sex practice [15]. Due to the differences in condom use between the two groups, we assume that the condom use beliefs of the general MSM population might be different from the HIV-positive population. To provide scientific evidence for the development of targeted interventions among general MSM, we investigated the prevalence of and the multidimensional belief factors associated with condom use with regular and nonregular partners in Sichuan, a province in China where the HIV prevalence rate of MSM is growing rapidly [16, 17].

## 2. Participant and Methods

### 2.1. Sampling and Recruitment

Participants in the study were recruited through snowball sampling, which was conducted in Sichuan, China, from November 2018 to April 2019. The inclusion criteria were (1) aged 16 or older, (2) having engaged in anal sex with males of any age, and (3) having lived in Sichuan Province for the past 3 months.

There were 35 cities in Sichuan Province in 2018. According to the information provided by the Sichuan Provincial Center for Disease Control and Prevention, 35 cities were categorized into high, medium, and low layers based on the estimated absolute number of MSM people. Then, in the three layers, we randomly selected one city as the research site, respectively. We firstly hired 15 investigators (9 from social communities and 6 from colleges) via a number of approaches, including gay communities, universities, and clubs. Then, 15 investigators were invited to participate in the training for the survey. Prospective participants were then recruited by the trained investigators; online (e.g., gay dating apps, such as *blued*, and social networking web sites, such as *microblog*) and offline (e.g., voluntary consultation testing clinics, bars, and teahouses). For online recruitment, the investigators posted the recruitment information on the chatting platforms of the social software or chat groups. Interested participants could chat with the investigators through the private chat window (social media programs, such as *blued* which only shows the nickname, age, height, weight, and place of residence). For offline recruitment, given that 9 investigators were volunteers from 2 MSM communities, in addition to daily testing services at workrooms, they visited gay bars (usually on Saturday and Sunday) and other gay sites (such as the teahouses and groves) three times a week to provide information on HIV and to provide testing services. They advertised the survey on site and invited people to participate. Those who showed interest and accepted to participate in the survey provided detailed informed consent. Guarantees were made on anonymity and their right to quit at any time without being questioned. Online questionnaires were subsequently sent to prospective participants, and investigators provided one-on-one interviews for offline participants (about 20 min). All participants were provided 30 Chinese RMB (about US $4) as compensation for their time. In addition, offline participants were additionally provided with free condoms and lubricants by the investigators.

**Figure 1:**
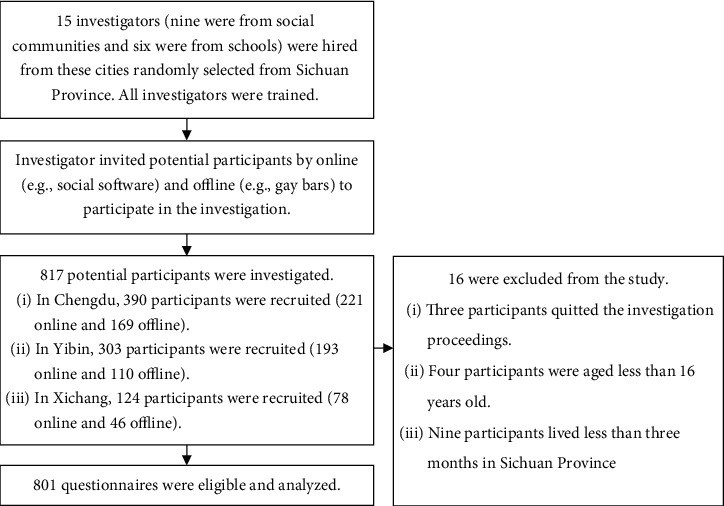
Sampling flowchart.

Eligible participants were screened according to the questionnaire filled in after survey. All participants under 16 years old or who had no sex with males were excluded from the study. All prospective participants provided written informed consent by electronic or pen signature. A total of 817 questionnaires were screened, of which 801 met the inclusion criteria and were analyzed. See the sampling flowchart for specific information (Figure 1). This study was approved by the ethics committee of the West China School of Public Health and the West China Fourth Hospital and was conducted in accordance with the Declaration of Helsinki (1964)/1964 Declaration of Helsinki.

### 2.2. Questionnaire Design and Contents

The questionnaire was designed by a panel consisting of two epidemiologists, one sociologist, one health psychologist, and four staff members from gay health and culture communities in Sichuan Province. The questionnaire was tested by ten eligible MSM and revised and finalized based on their feedback and panel discussion.

#### 2.2.1. Sociodemographic and AIDS-Related Characteristics

The sociodemographic characteristics included age, educational level, sexual orientation, marital status, vocation, monthly income, previous STD infection, and HIV infection status. For online recruitment, the HIV status of the participants was self-reported, while for offline recruitment, it was either self-reported or based on the result of voluntary tests provided for offline recruitment.

#### 2.2.2. Sexual Behaviors and Condom Use

Sexual behaviors referred to two types: (1) anal sex with regular partners, which was specifically defined as having had anal sex with male sexual partners in the last 6 months, and the sexual or romantic relationship should have lasted over 3 months (e.g., a couple with a sexual relationship). (2) Anal sex with nonregular partners, which was specifically defined as having had anal sex with male sexual partners in the last 6 months, and the sexual or romantic relationship should have lasted less than 3 months (e.g., one-night stand). Then, all participants were asked: “Have you had anal sex with regular partners in the last 6 months?” and “Have you had anal sex with non-regular partners in the last 6 months?”, with exemplified definition notes or interpretations from investigators.

For both types of sexual behaviors, we asked about condom use behavior with the question: “Have you used condoms during anal sex with regular (or non-regular) partners?” The answers consisted of four categories of frequency (“never,” “seldom,” “usually,” and “always”). The answer of “always” was considered as consistent condom use, and the other three categories were considered as inconsistent condom use.

#### 2.2.3. Dimensions of HBM Scale

The HBM scale comprised five dimensions, and each dimension was taken as a separate subscale. These dimensions included (1) the Perceived Threats Scale with two items (e.g., perceived risk of contracting AIDS in the next 6 months). Response categories of both items ranged from “very low” to “very high” and were scored from 1-5. (2) The Perceived Benefits Scale with two items (e.g., condom use would reduce your risk of HIV infection or other STDs). Response categories of both items ranged from “strongly disagree” to “strongly agree” and were scored from 1-5. (3) The Perceived Barriers Scale with two items (e.g., condom use would reduce sexual pleasure). Response categories of both items ranged from “strongly disagree” to “strongly agree” and were scored from 1-5. (4) The Self-efficacy Scale with five items (e.g., confidence in consistent condom use during anal sex). Response categories of each item ranged from “extremely lacking in confidence” to “extremely confident” and were scored from 1-5. (5) The Cues to Action Scale with two items of external cues (e.g., people around you remind you to use condoms, especially with nonregular partners) and two items of internal cues (e.g., you feel satisfied when using condoms during anal sex). Response categories of each item ranged from “strongly disagree” to “strongly agree” and were scored from 1-5. For the item “Who do you think is responsible for condom use during anal sex”, the answers of “your partner,” “both yourself and your partner,” and “yourself” were scored 1, 3, and 5, respectively. The value of internal reliability (Cronbach's alpha) of the HBM scale was 0.688.

### 2.3. Statistical Analysis

Consistent condom use with regular partners and nonregular partners was chosen as the dependent variables (0 = inconsistent condom use, 1 = consistent condom use). First, univariate logistic regression models were employed to estimate the effect of sociodemographic characteristics, AIDS-related characteristics, and dimensions of HBM on consistent condom use to obtain the univariate odds ratios (ORu). Sociodemographic and AIDS-related characteristics that were marginally significant (*P* < 0.1) in the univariate models were used as covariates. Second, the dimensions of HBM were separately entered into a single multivariate logistic regression model, which also comprised all the covariates, to obtain the single multivariate odds ratios (ORm). Finally, a summary multivariate model comprising all dimensions of HBM marginally significant (*P* < 0.1) in the univariate models and all the covariates was used to obtain the summary multivariate odds ratios (ORM). 95% confidence intervals (CI) were calculated. The subgroup analysis among HIV-negative MSM was employed, and the analysis was conducted in the same way.

All data were input by EpiData, version 3.1 (The EpiData Association, Odense, Denmark) for windows. The data analysis was implemented in SPSS, version 23.0 (SPSS Inc., Chicago, IL, USA) for windows. *P* < 0.05 was considered statically significant, and *P* < 0.1 was considered marginally significant.

## 3. Results

### 3.1. Sociodemographic and AIDS-Related Characteristics

Of the 801 participants in our questionnaire, 48.2% were less than 25 years old, 53.2% had received high school/technical school education, and 64.3% were identified as homosexual. 61.7% of the participants were unmarried, and 46.2% of them received a moderate monthly income of less than 2000 RMB (48.1% of them were students). For AIDS-related characteristics, 13.2% of participants had had previous STD infections, and 6.2% of them were diagnosed with HIV (Table 1).

### 3.2. Sexual Behaviors and Condom Use

Participants who had had anal sex with regular and nonregular partners were 39.1% and 53.6%, respectively. However, only 56.5% of them had consistently used condoms with regular partners, and only 60% of them had consistently used condoms with nonregular partners (Table 1).

### 3.3. Beliefs Based on HBM

Regarding perceived threats, less than 10% of the two groups of participants (i.e., had anal sex with a regular partner; had anal sex with nonregular partner) perceived the risk of HIV infection in the next 6 months. Besides, 57.8% of the participants who had had anal sex with regular partners and 73.4% of participants who had had anal sex with casual partner acknowledged the risk of HIV infection without consistent condom use. In terms of the perceived benefits of consistent condom use, more than half of the participants in both groups agreed with these benefits. For the perceived benefits of consistent condom use, less than 40% of participants in both groups agreed with these barriers. For self-efficacy, over 60% of participants in both groups showed confidence in consistent condom use, condom availability, and persuading the partners to use condoms, etc. As for cues to action, nearly 80% of the participants in both groups reported that they had received the information on condom use from people around them. However, only 57.5% and 59.2% of the participants felt satisfied with condom use when they had had anal sex with regular and nonregular partners, respectively (Table 2).

### 3.4. HBM-Based Beliefs Associated with Consistent Condom Use with Regular Partners

When taking consistent condom use with regular partners as the dependent variable, we found that age and marital status were significant in the univariate models and were therefore taken as covariates (Table 3). For beliefs based on HBM, we found that perceived threats (ORu = 1.14), perceived barriers (ORu = 0.69), self-efficacy (ORu = 1.26), and cues to action (ORu = 1.44) were significantly associated with the dependent variable in the univariate models. These dimensions also showed a significant association with the dependent variable in the single multivariate models. In the final summary multivariate model, perceived threats (ORM = 1.28, 95% CI: 1.10, 1.49), perceived barriers (ORM = 0.70, 95% CI: 0.60, 0.82), self-efficacy (ORM = 1.23, 95% CI: 1.14, 1.32), and cues to action (ORM = 1.21, 95% CI: 1.02, 1.43) showed significant associations with the dependent variable. See Table 4 for specific information. The subgroup analysis among HIV-negative participants showed similar results (See Supplementary Table 1).

### 3.5. HBM-Based Beliefs Associated with Consistent Condom Use with Nonregular Partners

When taking consistent condom use with nonregular partners as the dependent variable, we found that all variables of sociodemographic characteristics were significant in the univariate models and were therefore taken as covariates (Table 3). For beliefs based on HBM, we found that perceived threats (ORu = 0.89), perceived barriers (ORu = 0.74), self-efficacy (ORu = 1.21), and cues to action (ORu = 1.60) were significantly associated with the dependent variable in the univariate models. Only perceived barriers, self-efficacy, and cues to action showed significant associations with the dependent variable in the single multivariate models. In the final multivariate model, perceived barriers (ORM = 0.77, 95% CI: 0.67, 0.89), self-efficacy (ORM = 1.22, 95% CI: 1.13, 1.32), and cues to action (ORM = 1.53, 95% CI: 1.30, 1.80) showed significant associations with the dependent variable (Table 4). The subgroup analysis among HIV-negative participants showed similar results (See Supplementary Table 1).

## 4. Discussion

A large number of HIV monitoring data systems in China and other countries showed that the failure on the prevention of HIV spread among the MSM population can be ascribed to inconsistent condom use [18, 19]. Therefore, the condom use of MSM is of concern. Our results found that only 56.5% of the participants had consistently used condoms with regular partners, and only 60.0% of the participants had consistently used condoms with nonregular partners.

Our findings are in line with a recent meta-analysis in China [7]. Also similar to other previous studies, our study proved the association between psychological factors and consistent condom use among MSM based on the effective theoretical frame of HBM [6, 10]. Furthermore, compared to previous studies [6, 10], our study provided more quantitative evidence on condom use of the general MSM population in China. In our study, perceived barriers, as well as self-efficacy and cues to action, are closely associated with consistent condom use for anal sex with both regular and nonregular partners. There are several explanations for these results. First, for perceived barriers, some MSM individuals might believe that condom usage would affect the trust of intimacy [20], same-sex identity [21], or lower sexual pleasure during UAI [22], as a result of which they would choose not to use condoms. Second, self-efficacy of condom use usually refers to the reward and proper use of condoms and to the communication with partners concerning condom use [23, 24]. MSM with a high self-efficacy of condom use might have strong confidence and awareness to maintain condom use behaviors during anal sex, whether with regular partners or nonregular partners. It is mentioned that some studies revealed that self-efficacy showed a mediating effect between other dimensions (e.g., perceived severity and perceived barrier) and the outcome behavior [25, 26]. That is to say, other dimensions might affect the behavior partly through self-efficacy. Third, cues to action are the external and internal triggers of condom use behavior among general MSM. According to the social capital theory, support and networked connections are crucial determinants for individuals within a common community to form certain health behaviors [27], which seems to be a prerequisite for external cues. With enough support, individuals could gain enough cues or reminders from their family members or friends to strengthen their consciousness of safe sex [28]. Moreover, inner cues to action (e.g., responsibility and degree of satisfaction) also play an important role. MSM should be advised not to abandon personal responsibility for condom use or fully depend on their partners to make decisions, which is also mentioned in a previous study [6]. Overall, these results suggest that the removal of obstructive beliefs, improvement of confidence in condom use, and more cues to action might be helpful to behavior maintenance of consistent condom use with both regular and nonregular partners among MSM.

Despite the robust nature of the observed significant findings, several of the null findings are worth discussing. Firstly, perceived threats had effects on consistent condom use with regular partners, while had no effects on consistent condom use with nonregular partners. Such differences suggest us about the importance of segmentation (an effective principle in social marketing) [6, 29]. Previous studies found that MSM who had sex with nonregular partners tended to have higher scores of sexual sensation seeking than those who only had sex with regular partners [30]. Therefore, we speculated that although some of them who had had sex with nonregular partners perceived the risk of HIV infection, they still take any chance to seek sexual pleasure and think that “it seems to make no difference without using condoms this time.” Interestingly, the previous study, which explained the condom use of HIV-positive MSM in Chengdu, showed the association between perceived threats and condom use with nonregular partners [6]. Conceivably, of the HIV-positive MSM, some who perceived the risk of HIV transmission tended to use condoms. It may be necessary to further strengthen risk awareness education for general MSM groups.

Secondly, perceived benefits had no effect on consistent condom use with regular partners or nonregular partners. A possible explanation is that individuals' belief in the benefits of consistent condom use is overwhelmed by perceived barriers (e.g., condom use would reduce sexual pleasure) [10]. The perceived benefits of safe sex were not strong enough to encourage them to use condoms as a persistent behavior, while the perceived barriers (e.g., condoms affect sexual pleasure) granted them a chance to engage in unprotected anal sex, even if only once or twice. A previous study focused on HIV-positive MSM did not find the effect of perceived benefits either. However, perceived barriers were proven to be closely associated with condom use, especially between regular partners [6]. A qualitative study suggested that newly diagnosed HIV-positive MSM might find hinder in their ability to negotiate condom use with their regular partners to avoid breakup [31]. Combined with our results, we speculated this might be a common barrier also found in general MSM. Counseling concerning building/maintaining relationships and improving communication skills might be potentially useful [6]. It is mentioned that our subgroup analysis among HIV-negative MSM showed similar results to the general MSM, which might help confirm that some scales have more predictive power.

The current behavior interventions or services for HIV prevention among MSM mainly focus on providing health packages, such as condoms and AIDS testing kits, videos, and digital interventions (e.g., using apps) [32, 33]. However, if the scope of behavior interventions excluded the psychological factors, it would be difficult to achieve the intervention goals. According to our results, taking same-sex partners as the education object to reduce relationship barriers related to condom use might be helpful to reinforce sexual safety responsibility regulations [34]. Furthermore, it is important to improve and increase the comfort of condom [35]. Providing enough cues to action and raising social and family care and peer education might be effective strategies [36]. Also, in addition to the HBM, there are also other theories, such as the Theory of Planned Behavior (TPB) [37], the Information-Motivation-Behavioral skills model (IMB) [38], and other health behavior models [39]. These models have common strengths and unique contributions to understanding condom use behavior.

Several limitations should be considered in this study. First, our questionnaire was self-designed according to the frames of HBM and was not fully verified by its reliability and validity. However, our items were designed to be possibly suitable for Chinese MSM. Second, the study was carried out in a city in China with a relatively high HIV prevalence; caution should be taken when generalizing the findings. Besides, the findings, to some degree, can only be generalized to the general MSM who had a regular partner or nonregular partners. For other subgroups, such as those who had both regular and nonregular partners, more studies should be invited. Third, the concealed characteristics of the MSM population make the unavailability of random sampling, and nonprobability sampling leads to a certain selection bias. Fourth, this research collected information in the form of self-filled questionnaires. The respondents may have concealed part of the real information due to personal privacy and social expectations, or they could not accurately recall the problems involved in the questionnaire, which might have led to an information bias.

## 5. Conclusion

Inconsistent condom use behaviors with regular and nonregular partners are commonly observed in the MSM population of the Sichuan Province, China. Based on HBM, among this population, perceived barriers, self-efficacy, and cues to action were significantly associated with consistent condom use during anal sex with regular and nonregular partners. More attention should be focused on how to decrease the obstructive factors of condom use, how to improve the confidence of condom use, and how to lay out more cues to action to promote consistent condom use behavior.

## Figures and Tables

**Table 1:** Basic information of participants (*N* = 801).

Variables	*n*	%
Demographics		
Age		
<25	386	48.2
25-49	190	23.7
≥50	225	28.1
Education level		
Primary school or lower	218	27.2
Middle school	113	14.1
High school/technical school	426	53.2
College and above	44	5.5
Sexual orientation		
Homosexual	515	64.3
Bisexual/heterosexual	286	35.7
Marital status		
Unmarried	494	61.7
Married	230	28.7
Divorced/widowed	77	9.6
Vocation		
Employed	284	35.5
Unemployed/retired	132	16.5
Student	385	48.0
Monthly income (in RMB)		
<1000	153	19.1
1000-1999	217	27.1
2000-2999	133	16.6
3000-3999	114	14.2
≥4000	184	23.0
AIDS-related characteristic		
Previous STD infection		
Yes	106	13.2
No/unsure	695	86.8
HIV infection		
Yes	50	6.2
No/unsure	751	93.8
Sexual behavior		
Had anal sex with regular male sexual partner(s)		
Yes	313	39.1
No	488	60.9
Condom use with regular partner(s) (*n* = 313)		
Yes	177	56.5
No	136	43.5
Had anal sex with nonregular male sexual partner(s)		
Yes	429	53.6
No	372	46.4
Condom use with nonregular partners (*n* = 429)		
Yes	257	60.0
No	172	40.0

Notes: STD: “sexually transmitted diseases;” RMB: “renminbi;” 1 USD = 6.99 RMB at the time of survey.

**Table 2:** Health beliefs to condom use of participants.

	Had anal sex with regular partners (*n* = 313) %/M (SD)	Had anal sex with non-regular partners (*n* = 429) %/M (SD)
Perceived threats (% high/very high)		
Perceived risk of contracting AIDS in the next 6 months	9.3	9.3
Perceived risk of contracting AIDS if condomless anal sex occurred in the next 6 months	57.8	73.4
Perceived threats scale score	5.2 (2.0)	5.9 (1.8)
Perceived benefits of condom use (% agree/strongly agree)		
Condom use would reduce your risk of HIV infection or other STDs	54.3	64.8
Condom use would make you feel more at ease and no longer regret	74.4	82.1
Perceived benefits scale score	7.4 (1.7)	7.8 (1.7)
Perceived barriers toward condom use (% agree/strongly agree)		
Condom use would reduce sexual pleasure	24.3	32.9
Condom use would damage your intimate relationship with your male partner	31.9	38.9
Perceived barriers scale score	5.4 (2.1)	5.7 (2.1)
Self-efficacy toward consistent condom use (% confident/extremely confident)		
Confidence in consistent condom use during anal sex	76.0	84.6
Confidence in condom availability during anal sex	71.2	79.7
Confidence in persuading the partner to use condoms even if he does not want to	72.8	77.9
Confidence in refusing to have condomless anal sex with mutually affectionate males	70.0	68.3
Confidence in refusing to have condomless anal sex with males who have been in contact for some time	63.3	65.7
Self-efficacy scale score	19.8 (4.7)	20.3 (3.9)
Cues to action		
People around you remind you to use condoms, especially with non-regular partners (% agree/strongly agree)	86.9	79.3
People around you remind you that condoms are not just for contraception (% agree/strongly agree)	85.3	82.8
You feel satisfied to use condoms during anal sex (% agree/strongly agree)	57.5	59.2
Who do you think is responsible for condom use during anal sex (% yourself/both yourself and your partner)	96.8	86.1
Cues to action scale score	12.3 (1.5)	12.2 (1.5)

Notes: M: mean; SD: Standard deviation.

**Table 3:** Single analyses between demographics and consistent condoms use with regular and nonregular partner.

	Consistent condoms use with regular partner (*n* = 313) ORu (95% CI)	Consistent condoms use with nonregular partner (*n* = 429) ORu (95% CI)
Demographics		
Age		
<25	1.00	1.00
25-49	0.79(0.46, 1.37)	0.56 (0.34, 0.95)^∗^
≥50	0.42 (0.23, 0.76)^∗∗^	0.20 (0.13, 0.33)^∗∗^
Education level		
Primary school or lower	1.00	1.00
Middle school	1.24 (0.54, 2.87)	1.45 (0.80, 2.63)
High school/technical school	1.60 (0.87, 2.95)	4.82 (3.02, 7.68)^∗∗^
College and above	2.24 (0.82, 6.12)	3.73 (1.37, 10.18)^∗^
Sexual orientation		
Homosexual	1.00	1.00
Bisexual/heterosexual	0.78 (0.46, 1.32)	0.43 (0.29, 0.64)^∗∗^
Marital status		
Unmarried	1.00	1.00
Married	0.56 (0.32, 0.99)^∗^	0.23 (0.15, 0.37)^∗∗^
Divorced/widowed	0.22 (0.09, 0.55)^∗∗^	0.26 (0.14, 0.47)^∗∗^
Vocation		
Employed	1.00	1.00
Unemployed/retired	0.54 (0.21, 1.39)	0.33 (0.19, 0.58)^∗∗^
Student	1.38 (0.86, 2.22)	2.40 (1.51, 3.79)^∗∗^
Monthly income (in RMB)		
<1000	1.00	1.00
1000-1999	0.92 (0.47, 1.79)	2.15 (1.20, 3.85)^∗^
2000-2999	1.72 (0.74, 1.96)	1.92 (1.02, 3.62)^∗^
3000-3999	0.57 (0.24, 1.39)	0.75 (0.38, 1.45)
≥4000	0.75 (0.38, 1.48)	1.75 (0.96, 3.18)^†^
AIDS-related characteristic		
Previous STD infection		
Yes	1.00	1.00
No/unsure	1.65 (0.81, 3.34)	1.43 (0.85, 2.39)
HIV infection		
Yes	1.00	1.00
No/unsure	1.63 (0.68, 1.89)	1.80 (0.85, 3.79)

Note: STD: “sexually transmitted diseases.” ORu: univariate odds ratios. ^†^0.05 < *P* < 0.1; ^∗^*P* < 0.05; ^∗∗^*P* < 0.01.

**Table 4:** Association between dimensions of HBM and consistent condoms use with regular and nonregular partner.

	Consistent condoms use with regular partners (*n* = 313)			Consistent condoms use with nonregular partners (*n* = 429)		
	ORu (95% CI)	ORm (95% CI)^a^	ORM (95% CI)	ORu (95% CI)	ORm (95% CI) ^b^	ORM (95% CI)
Perceived threats scale	1.14 (1.01, 1.27)^∗^	1.20 (1.06, 1.36)^∗∗^	1.28 (1.10, 1.49)^∗∗^	0.80 (0.71, 0.89)^∗∗^	0.89 (0.79, 1.01)^†^	0.97 (0.83, 1.13)
Perceived benefits scale	1.01 (0.88, 1.15)	1.07 (0.93, 1.24)	—	0.91 (0.81, 1.02)	1.16 (1.00, 1.34)^†^	—
Perceived barriers scale	0.69 (0.60, 0.78)^∗∗^	0.69 (0.60, 0.78)^∗∗^	0.70 (0.60, 0.82)^∗∗^	0.74 (0.67, 0.82)^∗∗^	0.71 (0.63, 0.79)^∗∗^	0.77 (0.67, 0.89)^∗∗^
Self-efficacy scale	1.26 (1.18, 1.34)^∗∗^	1.29 (1.20, 1.39)^∗∗^	1.23 (1.14, 1.32)^∗∗^	1.21 (1.14, 1.28)^∗∗^	1.29 (1.20, 1.39)^∗∗^	1.22 (1.13, 1.32)^∗∗^
Cues to action scale	1.44 (1.26, 1.64)^∗∗^	1.34 (1.35, 1.82)^∗∗^	1.21 (1.02, 1.43)^∗^	1.60 (1.41, 1.81)^∗∗^	1.78 (1.54, 2.07)^∗∗^	1.53 (1.30, 1.80^∗∗^

Note: ORu: univariate odds ratios; ORm: single multivariate odds ratio; ORM: summary multivariate odds ratios. ^a^ include one dimension of HBM and variables of sociodemographic and AIDS-related characteristics marginally significant (*P* < 0.1) in univariate analysis (i.e., age and marital status). ^b^ include one dimension of HBM and variables of sociodemographic and AIDS-related characteristics marginally significant (*P* < 0.1) in univariate analysis (i.e., age, educational level, sexual orientation, marital status, vocation, and monthly incomes). ^†^0.05 < *P* < 0.1; ^∗^*P* < 0.05; ^∗∗^*P* < 0.01.

## Data Availability

The data was collected by experienced scientists and health personnel. The datasets used and/or analyzed during the current study are available from the corresponding author on reasonable request.

## Supplementary Materials

Supplementary Materials Supplement Table 1: subgroup analysis among the HIV-negative MSM.
