# Supplementary material for: Association between Psychological Factors and Condom Use with Regular and Nonregular Male Sexual Partners among Chinese MSM: A Quantitative Study Based on the Health Belief Model
Source: Biomed Res Int. 2020 Sep 28;2020:5807162. doi: 10.1155/2020/5807162 (PMC7539081; doi:10.1155/2020/5807162)
Supplement: Supplementary Materials — Supplement Table 1: subgroup analysis among the HIV-negative MSM. [file 5807162.f1.doc]

**Supplement Table 1. Sub-group analysis among the HIV-negative MSM**

|  | Consistent condoms use with regular partners (n=291) | | | Consistent condoms use with non-regular partners (n=399) | | |
| --- | --- | --- | --- | --- | --- | --- |
|  | ORu (95%CI) | ORm (95%CI) ^a^ | ORM (95%CI) | ORu (95%CI) | ORm (95%CI) ^b^ | ORM (95%CI) |
| Perceived Threats Scale | 1.16 (1.02, 1.32) * | 1.22 (1.07, 1.41) ** | 1.28 (1.09, 1.51) ** | 0.73 (0.64, 0.84) ** | 0.84 (0.73, 0.98) * | 0.94 (0.79, 1.13) |
| Perceived Benefits Scale | 0.99 (0.86, 1.42) | 1.06 (0.91, 1.24) | — | 0.87 (0.77, 0.99) * | 1.13 (0.97, 1.32) | 1.02 (0.84, 1.24) |
| Perceived Barriers Scale | 0.67 (0.59, 0.77) ** | 0.67 (0.58, 0.77) ** | 0.71 (0.61, 0.84) ** | 0.73 (0.65, 0.81) ** | 0.70 (0.62, 0.79) ** | 0.78 (0.67, 0.91) ** |
| Self-efficacy Scale | 1.28 (1.19, 1.38) ** | 1.33 (1.23, 1.45) ** | 1.26 (1.16, 1.38) ** | 1.22 (1.15, 1.30) ** | 1.36 (1.25, 1.48) ** | 1.27 (1.16, 1.39) ** |
| Cues to Action Scale | 1.36 (1.19, 1.56) ** | 1.50 (1.29, 1.76) ** | 1.12 (0.94, 1.34) | 1.54 (1.36, 1.76) ** | 1.73 (1.48, 2.02) ** | 1.44 (1.21, 1.71) ** |

Note: ORu, univariate odds ratios; ORm, single multivariate odds ratio, ORM, summary multivariate odds ratios.

^a^: include one dimension of HBM and variables of sociodemographic and AIDS-related characteristics marginally significant (*P*<0.1) in univariate analysis (i.e., age, marital status, vocation).

^b^: include one dimension of HBM and variables of sociodemographic and AIDS-related characteristics marginally significant (*P*<0.1) in univariate analysis (i.e., age, educational level, sexual orientation, marital status, vocation, monthly incomes).

† 0.05<*P*<0.1; * *P*<0.05; ** *P*<0.01
